# Microtubule Inner Protein CFAP77 Contributes to Sperm Motility and Male Fertility in Mice

**DOI:** 10.1111/andr.70152

**Published:** 2025-11-29

**Authors:** Haoting Wang, Anh Hoang Pham, Mengjiao Luo, Masahito Ikawa, Haruhiko Miyata

**Affiliations:** ^1^ Research Institute for Microbial Diseases The University of Osaka Suita Osaka Japan; ^2^ Graduate School of Pharmaceutical Sciences The University of Osaka Suita Osaka Japan; ^3^ Graduate School of Medicine The University of Osaka Suita Osaka Japan; ^4^ Center For Infectious Disease Education and Research The University of Osaka Suita Osaka Japan; ^5^ Center For Advanced Modalities and DDS (CAMaD) The University of Osaka Suita Osaka Japan; ^6^ The Institute of Medical Science The University of Tokyo Minato‐ku Tokyo Japan

**Keywords:** male infertility, Microtubule inner proteins, sperm motility

## Abstract

**Background:**

Sperm motility is essential for male fertility, and its regulation is dependent on the structural integrity of the axoneme. The axoneme consists of a conserved “9+2” microtubule arrangement and is supported by microtubule inner proteins. However, the functional significance of many microtubule inner proteins remains unclear. Cilia‐ and flagella‐associated protein 77 (CFAP77) has been identified as a microtubule inner protein in various species, but its role in mammalian sperm function has not been fully elucidated.

**Objectives:**

This study aims to investigate the function of CFAP77 in sperm motility and male fertility using a *Cfap77* knockout mouse model.

**Materials and Methods:**

*Cfap77* knockout mice were generated using the CRISPR‐Cas9 system. Male fertility was assessed by mating tests, and sperm motility was analyzed using computer‐assisted sperm analysis. Immunoblotting and immunoprecipitation‐mass spectrometry were performed to determine CFAP77's localization and interaction with other microtubule inner proteins.

**Results:**

We found that CFAP77 localized to the sperm flagella in mice. Moreover, *Cfap77* knockout males exhibited significantly reduced fertility, with impaired sperm motility despite normal morphology. Immunoprecipitation‐mass spectrometry analysis revealed that CFAP77 interacts with TEKTL1, and CFAP77 loss leads to a reduced amount of TEKTL1 in spermatozoa.

**Discussion and Conclusion:**

We demonstrate that CFAP77 is crucial for sperm motility and male fertility. The interaction between CFAP77 and other microtubule inner proteins suggests a role in stabilizing other microtubule inner proteins and regulating flagellar function. These results provide insights into the molecular mechanisms of sperm motility regulation.

## 1 Introduction

Sperm motility is essential for male fertility as spermatozoa must travel through the female reproductive tract to reach and fertilize the egg [1]. This motility is driven by the sperm flagellum, a whip‐like structure that generates propulsion through coordinated bending and beating [2]. Impaired flagella lead to reduced sperm motility, which is a major cause of male infertility in both humans [3] and mice [4]. Understanding the molecular mechanisms that regulate sperm motility is therefore crucial for deepening the understanding of male reproductive health.

**FIGURE 1:**
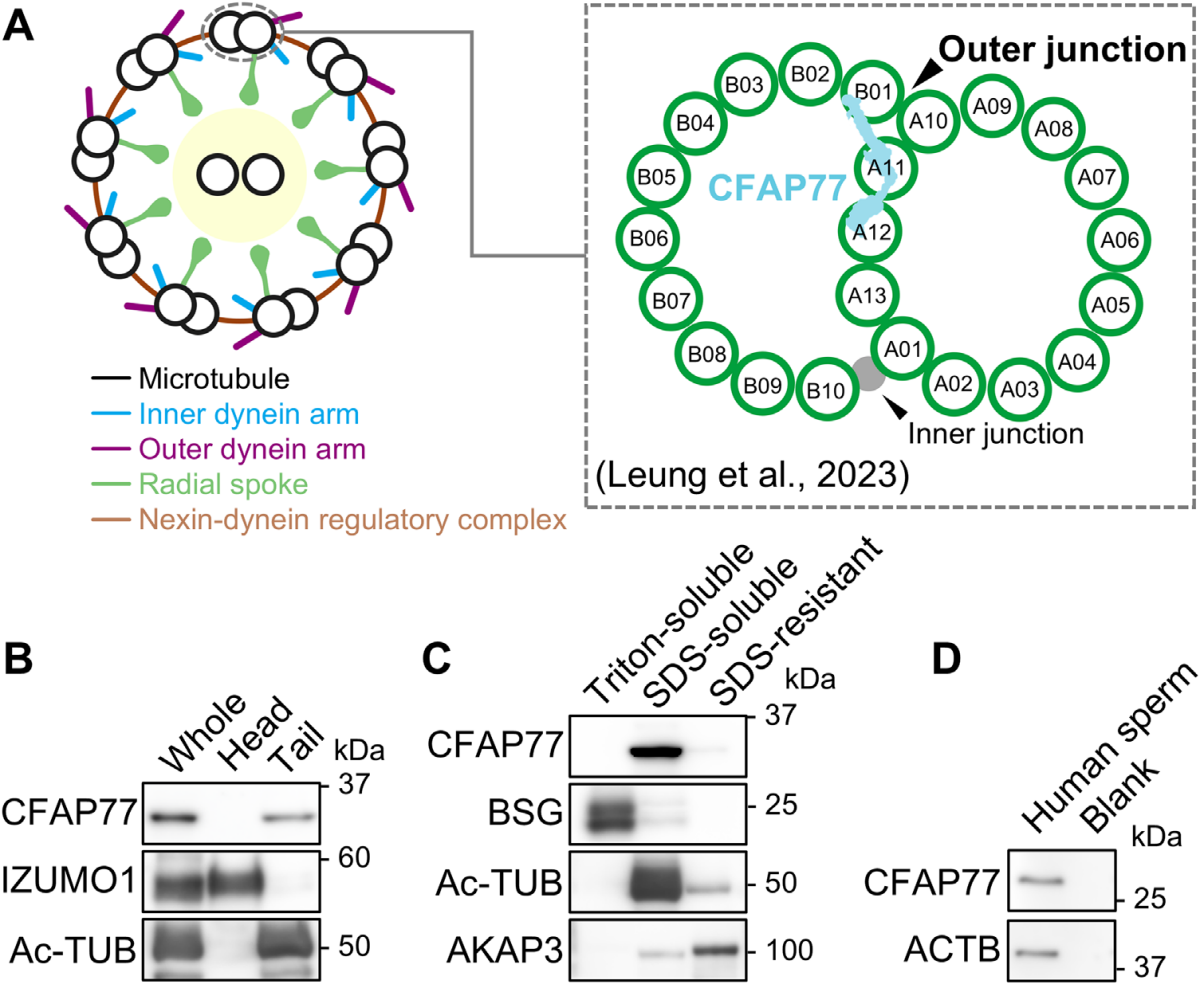
CFAP77 is evolutionarily conserved and localized in mouse sperm tails. (A) Schematic diagram of cross‐sectional views of sperm axoneme and microtubule doublets. (B) Immunoblotting was performed using sperm head‐tail separation lysates. IZUMO1 and acetylated tubulin (Ac‐TUB) mark the successful separation of sperm heads and tails, respectively. (C) Immunoblotting was performed using sperm fractionation lysates. BASIGIN (BSG), Ac‐TUB, and AKAP3 represent the successful fractionation. (D) Immunoblot analyses using human spermatozoa. Beta‐actin (ACTB) was used as a loading control.

The structural backbone of the sperm flagellum is the axoneme, a highly conserved microtubule‐based scaffold found in cilia and flagella across species [5]. The axoneme is composed of a signature “9+2” arrangement of microtubules, consisting of nine outer doublets surrounding a central pair singlet. On each microtubule doublet, inner dynein arms, outer dynein arms, and radial spokes are decorated, and nine microtubule doublets are connected by nexin‐dynein regulatory complexes (Figure 1A). These accessory structures all contribute to maintaining normal flagellar motility [4, 6, 7]. Of note, microtubule doublets consist of a complete tubule (A‐tubule) and an incomplete tubule (B‐tubule), in which microtubule protofilaments can be labeled as A01‐A13 and B01‐B10, respectively (Figure 1A) [8]. The inner junction of the A‐tubule and B‐tubule is linked by CFAP20 [9, 10] and PACRG [11, 12], while the outer junction is thought to be formed by a direct interaction between protofilaments. Recent advances in cryo‐electron microscopy (cryo‐EM) have provided unprecedented insights into the molecular architecture of the axoneme by revealing the presence of microtubule inner proteins (MIPs) [13, 14, 15, 16]. MIPs localize within the microtubule lumen and are thought to stabilize microtubule doublets, regulating sperm motility [17, 18]. Despite these insights, the functional significance of many MIPs has yet to be fully elucidated, and their specific roles in sperm motility remain to be determined.

CFAP77 is a MIP that has been detected in the axoneme of the sea urchin, bovine [13], and mouse spermatozoa [17] by cryo‐EM/‐electron tomography (ET), suggesting a possible role of CFAP77 in flagellar function. CFAP77 is located in the inner wall of the B‐tubule and is attached to A11, A12, and B01 protofilaments (Figure 1A), presenting every 16 nm localization along the microtubule doublets in bovine spermatozoa [13]. Another study proposed that CFAP77 is involved in ciliary motility and microtubule doublets’ stability in a unicellular organism, *Tetrahymena thermophila* [19], but direct experimental evidence towards the role of CFAP77 in mammalian spermatozoa was lacking.

In this study, we generated a *Cfap77* knockout (KO) mouse model to explore its role in mammalian sperm‐related function. We found that loss of CFAP77 significantly reduces sperm motility and leads to decreased male fertility in mice. Moreover, CFAP77 interacts with other MIPs, and the ablation of CFAP77 leads to the loss of MIPs. Our study highlights CFAP77 as a key player in axonemal integrity and sperm flagellar motility.

## 2 Materials and Methods

### 2.1 Animals

All animal experiments were conducted in accordance with ethical guidelines and were approved by the Animal Care and Use Committee at the Research Institute for Microbial Diseases, The University of Osaka (Approval numbers: #Biken‐AP‐H30‐01 and #Biken‐AP‐R03‐01). Mice were obtained from Japan SLC (Shizuoka, Japan) or CLEA Japan (Tokyo, Japan) and were housed in a specific‐pathogen‐free facility with ad libitum access to food and water under a controlled 12‐h light/dark cycle.

The generation of KO mice was carried out following previously established protocols [20]. Briefly, female B6D2F1 mice were superovulated via intraperitoneal administration of CARD HyperOva (Kyudo, Saga, Japan) and human chorionic gonadotropin (hCG) (ASKA Pharmaceutical, Tokyo, Japan), after which they were paired with wild‐type B6D2F1 males. Two‐pronuclear (2PN) zygotes were subsequently collected from the female mice. The 2PN zygotes were then subjected to electroporation with a ribonucleoprotein complex consisting of CAS9 (#A36497, Thermo Fisher, Waltham, MA), CRISPR RNA (crRNA) (Sigma‐Aldrich, St. Louis, MO), and trans‐activating crRNA (tracrRNA) (#TRACRRNA05N‐5NMOL, Sigma‐Aldrich). The sequences of guide RNAs and PCR primers for genotyping are listed in Table S1. *Cfap77* KO mice generated in this study will be made available at the RIKEN BioResource Research Center (Ibaraki, Japan) and the Center for Animal Resources and Development, Kumamoto University (Kumamoto, Japan).

### 2.2 Mating Test

Each sexually mature KO or wild‐type (WT) male was housed with three 7‐week‐old females for a duration of 8 weeks. During this period, vaginal plugs were monitored as an indication of mating, and the number of offspring was recorded. After the 8‐week cohabitation, the males were separated from the cages, and pup counts continued for an additional 3 weeks to account for the completion of the final litters by the females.

### 2.3 Histological Study

To fix the testes and epididymis, Bouin's fluid (#16045‐1, Polysciences, Inc., Warrington, PA) was used. The samples were then processed for paraffin embedding, and 5 µm sections were prepared. The sections were treated with 1% periodic acid (Nacalai Tesque, Kyoto, Japan) for 10 min, followed by incubation with Schiff's reagent (Wako, Osaka, Japan) for 20 min. After thorough rinsing with tap water, the slides were counterstained with hematoxylin for 1 min and subsequently visualized with an Olympus BX‐53 microscope (Tokyo, Japan).

### 2.4 Immunohistochemistry

Immunohistochemistry was performed as previously described with slight modifications [21]. Tissues were fixed with 4% PFA (Thermo Fisher Scientific, Waltham, MA) and sectioned. Tissue sections were permeabilized with 0.1% Triton X‐100 (Nacalai Tesque) and blocked with 1% bovine serum albumin (BSA; Sigma‐Aldrich). Then, specimens were probed with primary antibody overnight, and probed with secondary antibody for 2 h after a quick wash. Nuclei were labeled by Hoechst33342 (Thermo Fisher Scientific), and slides were mounted by Epredia Immu‐Mount (Thermo Fisher Scientific). Slides were observed by an Olympus BX‐53 microscope or a Nikon C2 Eclipse Ti microscope (Tokyo, Japan).

### 2.5 Immunoblotting

Immunoblotting was performed as previously described [22]. In brief, lysates in sodium dodecyl sulfate (SDS) sample buffer were loaded onto SDS‐PAGE gels (ATTO, Osaka, Japan) and transferred onto polyvinylidene fluoride (PVDF) membranes. Then, membranes were blocked, probed with primary antibodies, and subsequently probed with secondary antibodies. The band detection was performed using the Amersham ImageQuant 800 system (Cytiva, Tokyo, Japan). Lysates for sperm head‐tail separation and sperm fractionation were prepared as previously described [23].

### 2.6 Antibodies

Antibodies used are listed in Table S2. Anti‐IZUMO1 and anti‐LRRC23 antibodies were established in the previous studies [24, 25].

### 2.7 Analysis of Sperm Motility

Sperm motility was assessed using the CEROS II sperm analysis system (Hamilton Thorne Biosciences, Beverly, MA) as in previous reports [26, 27]. Cauda epididymal spermatozoa were extracted into a 100 µL TYH drop [28]. Following incubation at 37°C with 5% CO_2_ for 10 min and 120 min, sperm motility was assessed.

### 2.8 Immunoprecipitation

Immunoprecipitation (IP) was conducted as previously described [29]. Testis and cell lysates were subjected to IP using Dynabeads magnetic beads (Thermo Fisher) in accordance with the manufacturer's instructions.

### 2.9 Mass Spectrometry

Immunoprecipitation samples were analyzed using nanocapillary reversed‐phase liquid chromatography (LC)‐mass spectrometry (MS)/MS. Separation was performed on a C18 column (IonOpticks, Victoria, Australia) integrated into a nanoLC system (Bruker Daltonics, Billerica, MA), coupled to a timsTOF Pro mass spectrometer (Bruker Daltonics, Billerica, MA) with a CaptiveSpray nano‐electrospray ion source (Bruker Daltonics, Billerica, MA). Raw data were processed using DataAnalysis software (Bruker Daltonics, Billerica, MA), and protein identification was carried out via MASCOT (Matrix Science, Tokyo, Japan) using the UniProt database. Quantitative values and fold changes were subsequently analyzed using Scaffold5 (Proteome Software, Portland, OR).

### 2.10 Collection of Human Spermatozoa Sample

Human spermatozoa were obtained from healthy donors. The experiments involving human samples were approved by the Ethics Committee of the Research Institute for Microbial Diseases, The University of Osaka. Human spermatozoa lysates were prepared as described previously [27].

### 2.11 Culture and Transfection of HEK293T Cells

HEK293T cells were maintained in DMEM (Thermo Fisher Scientific) supplemented with 10% fetal bovine serum (FBS) (Sigma‐Aldrich). Cells were transfected using the calcium phosphate method after reaching 60% confluency and cultured for 16 h. Then, the medium was changed to DMEM without FBS and further incubated for 2 days before performing downstream analyses.

### 2.12 Statistical Analysis

All data are expressed as mean ± standard deviation. All comparisons were performed using Student's t‐test. Differences were considered significant at ^*^ *p *< 0.05, ^**^ *p* < 0.01, and ^***^ *p* < 0.001.

## 3 Results

### 3.1 CFAP77 Is Evolutionarily Conserved and Associated With Sperm Axonemes

According to the data from Ensembl [30], *CFAP77* is evolutionarily conserved in various species, including humans, bovines, and mice (Figure S1A). When the amino acid sequences of human and mouse CFAP77 were compared, they showed high similarity along the sequences (Figure S1B). In adult mice, *Cfap77* shows expression in the lung and the testis (Figure S1C) [31]. In testes, haploid round spermatids are produced through meiosis, and subsequently, spermatozoa are formed through spermiogenesis, a process divided into 16 steps in mice and characterized by drastic morphological changes such as flagellar elongation and nuclear condensation [32]. By visualizing the single‐cell RNA sequencing data [33], we found that the expression of *Cfap77* starts from Step 1 spermatids, peaks at Step 3 spermatids, and then gradually decreases (Figure S1D), consistent with the timing of axonemal elongation [34]. To analyze the localization of CFAP77 in mouse spermatozoa, we performed immunoblotting on sperm head‐tail lysates (Figure 1B). CFAP77 showed signals in sperm tails but not in heads. Then, we performed immunoblotting on sperm fractionation lysates. We fractionated mature spermatozoa into Triton‐soluble, SDS‐soluble, and SDS‐resistant fractions, which mainly compose cytoplasmic/membrane‐associated proteins, axonemal proteins, and accessory structures’ proteins, respectively. The CFAP77 signal was found in the SDS‐soluble fraction, suggesting that CFAP77 is associated with sperm axonemes (Figure 1C). Further, we confirmed the presence of CFAP77 in human spermatozoa with immunoblotting (Figure 1D).

### 3.2 CFAP77 is Important for Male Fertility in Mice

**FIGURE 2:**
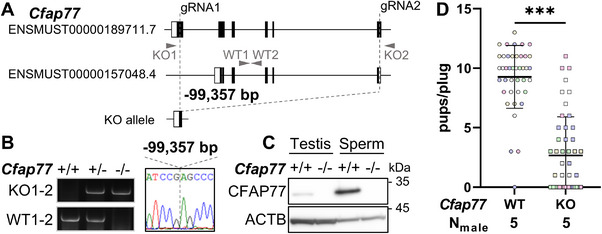
Generation and mating test of *Cfap77* KO male mice. (A) KO strategy of *Cfap77*. *Cfap77* possesses two splicing variants, and we used two guide RNAs (gRNAs) to target the longest transcript variant. Arrowheads indicate the locations of primers. (B) Left: genotyping of *Cfap77* mutant mice with PCR. Right: Deletion in the coding region of *Cfap77* was confirmed by Sanger sequencing. (C) The absence of CFAP77 in the testis and spermatozoa of *Cfap77* KO mice was confirmed by immunoblotting. Beta‐actin (ACTB) was used as a loading control. (D) Mating test of *Cfap77* KO male mice. *Cfap77* KO males exhibited significantly decreased fertility relative to WT males. Data from the same mice are coded in the same color. ^***^ *p* < 0.001.

To test if CFAP77 plays a role in male fertility, we generated *Cfap77* KO mice with the CRISPR‐Cas9 system (Figure 2A). We electroporated 90 zygotes and transplanted 74 two‐cell stage embryos into three pseudopregnant females. Out of seven pups obtained, one pup possesses a largely deleted allele of *Cfap77*. After the subsequent mating, by the cohabitation of one *Cfap77^+/−^* male with two *Cfap77^−/−^* females, we were able to obtain *Cfap77* KO offspring at an expected ratio (Figure S2A). No overt gross defects in development, behavior, or survival were found in *Cfap77* KO mice. We confirmed *Cfap77* KO mice with a 99,357‐base pair deletion in the coding region, which was detected by polymerase chain reaction (PCR) and subsequent Sanger sequencing (Figure 2B). At the protein level, CFAP77 is confirmed to be absent in the testis and cauda epididymal spermatozoa of *Cfap77* KO males by immunoblotting (Figure 2C). We then housed five KO males individually with females to test their fertility. As a result, *Cfap77* KO males were subfertile, which showed a significantly decreased number of pups per plug versus WT males (Figure 2D). Therefore, CFAP77 is important for maintaining normal male fertility in mice.

### 3.3 Ablation of *Cfap77* Results in Impaired Sperm Motility

**FIGURE 3:**
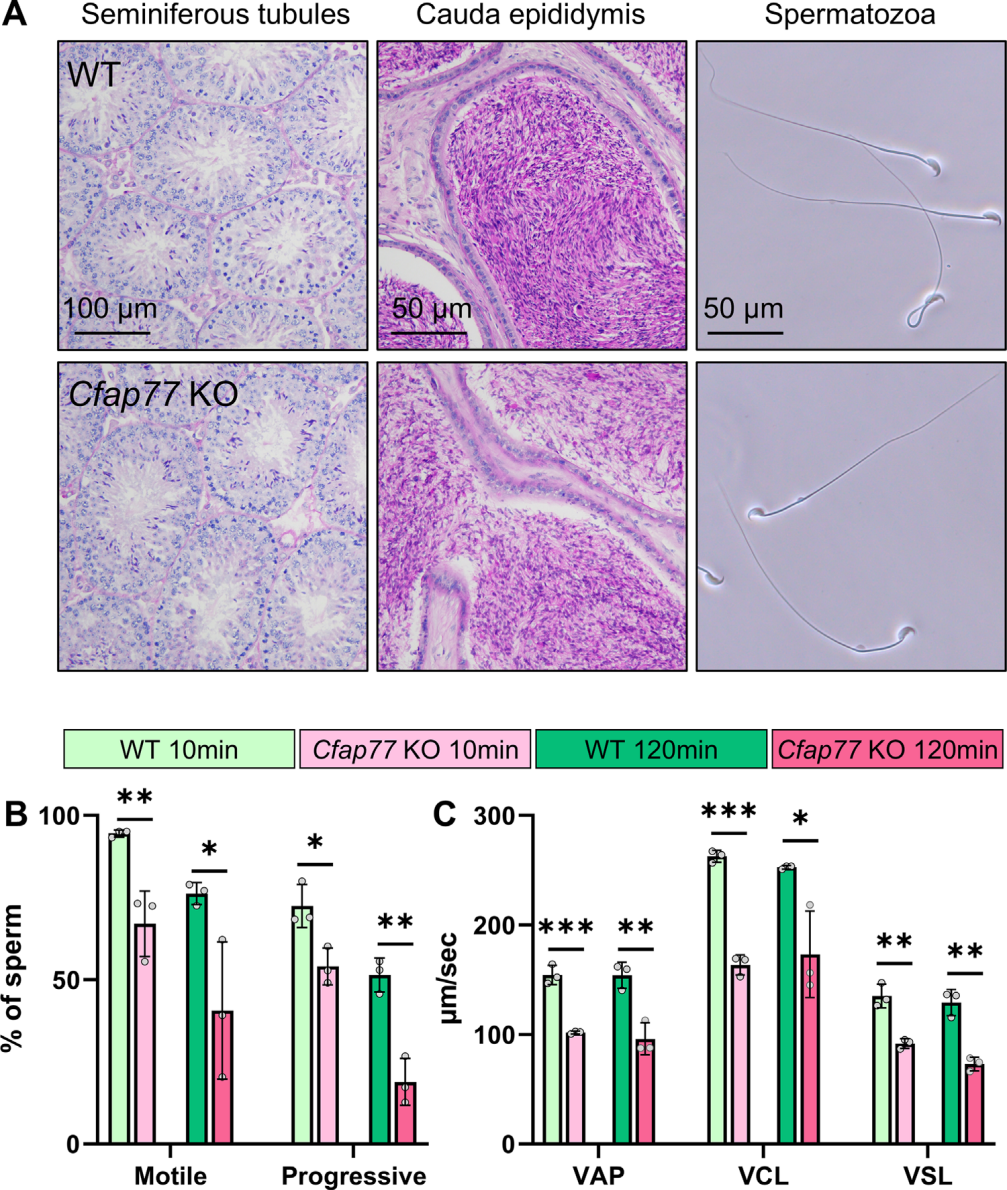
Morphological and motility analyses of *Cfap77* KO spermatozoa. (A) Morphological analyses of testicular sections, cauda epididymal sections, and cauda epididymal spermatozoa. (B) The percentages of motile and progressively motile spermatozoa in *Cfap77* KO mice. (C) Average path velocity (VAP), curvilinear velocity (VCL), and straight line velocity (VSL) of *Cfap77* KO spermatozoa. ^*^ *p* < 0.05, ^**^ *p* < 0.01, ^***^ *p* < 0.001.

We next made sections of male reproductive organs to study the causes of male subfertility in *Cfap77* KO mice. No abnormalities were found in the sections of the *Cfap77* KO testes and cauda epididymis, with a similar number of spermatozoa observed in the cauda epididymis between WT and *Cfap77* KO males (Figure 3A). Furthermore, the morphology of cauda epididymal spermatozoa from *Cfap77* KO males was indistinguishable from WT spermatozoa (Figure 3A). As *Cfap77* is also expressed in the lung, we validated whether the absence of CFAP77 affects ciliogenesis in tracheal cilia with immunohistochemistry using an anti‐acetylated tubulin antibody. Nevertheless, no overt abnormalities were found in the tracheal cilia of *Cfap77* KO mice (Figure S2B).

As there were no apparent anomalies in the reproductive organs or sperm morphology, we next studied the sperm motility of *Cfap77* KO males. As the movement pattern changes in spermatozoa accompanying capacitation [35], a process indispensable for reproduction [1], we measured sperm motility after 10 min and 120 min of incubation in a capacitation medium [28], corresponding to the motility of non‐capacitated and capacitated spermatozoa. As a result, the percentage of motile spermatozoa and progressively motile spermatozoa significantly decreased in both 10 and 120 min of incubation in *Cfap77* KO males (Figure 3B, Movie S1). Further, we measured sperm velocity parameters such as straight line velocity (VSL), curvilinear velocity (VCL), and average path velocity (VAP) in both 10‐ and 120‐min incubation. We found that all three parameters decreased despite the incubation time (Figure 3C), demonstrating that CFAP77 is vital for sperm motility.

To examine if the reduced sperm motility is caused by damaged integrity of axonemal components such as radial spokes and dynein arms, we performed immunohistochemistry on the *Cfap77* KO testicular sections. RSPH9, LRRC23, and DNAI2 were used to label radial spoke 1/2, radial spoke 3, and outer dynein arm, respectively [36, 37]. We found the signals of these proteins in the flagella of *Cfap77* KO testes, suggesting that these components may be formed correctly (Figure S3A–C).

### 3.4 CFAP77 Interacts With Sperm MIPs, and Loss of CFAP77 Reduces the Amounts of MIPs

**FIGURE 4:**
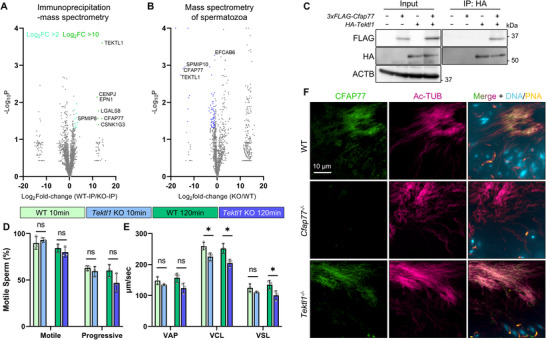
Absence of CFAP77‐associated TEKTL1 does not alter CFAP77 localization in sperm flagella. (A) Immunoprecipitation‐mass spectrometry study using an anti‐CFAP77 antibody. Each protein identified with *p* < 0.05 was colored according to the fold‐change of quantitative values. Immunoprecipitation products of WT to *Cfap77* KO testes were compared. (B) Mass spectrometry analyses of WT and *Cfap77* KO sperm lysates. Proteins significantly decreased in *Cfap77* KO spermatozoa were colored blue. (C) Immunoblot on immunoprecipitation product derived from HEK293T cells transfected with *3×FLAG‐Cfap77* and *HA‐Tektl1*. Beta‐actin (ACTB) was used as a loading control. (D) The percentages of motile and progressively motile spermatozoa in *Tektl1* KO mice. (E) Average path velocity (VAP), curvilinear velocity (VCL), and straight line velocity (VSL) of *Tektl1* KO spermatozoa. ^*^ *p* < 0.05. (F) Immunohistochemistry of WT, *Cfap77* KO, and *Tektl1* KO testes. CFAP77 was present in *Tektl1* KO testes, and its signal co‐localized with acetylated‐tubulin (Ac‐TUB).

To analyze the function of CFAP77, we performed immunoprecipitation with an anti‐CFAP77 antibody using testis lysates, followed by mass spectrometry. We used *Cfap77* KO testes with the same beads and antibody for the immunoprecipitation as a negative control. We found that CFAP77 interacts with sperm MIPs, TEKTL1, and SPMIP8 (Figure 4A, Table S3), with TEKTL1 showing a high fold‐change and low *p* value. This CFAP77‐TEKTL1 interaction is shown in the previous cryo‐EM study, as TEKTL1 forms filaments and binds to A11 and A12 protofilaments and CFAP77 [13]. We next questioned if TEKTL1 could be integrated into the flagella of mature spermatozoa in the absence of CFAP77, by performing mass spectrometry in the sperm lysates (Figure 4B, Table S4). The result indicates that TEKTL1 was significantly decreased in *Cfap77* KO spermatozoa, together with other MIPs such as EFCAB6 and SPMIP10 [13]. As TEKTL1 was downregulated in *Cfap77* KO spermatozoa, we further confirmed that CFAP77 interacts with TEKTL1 in HEK293T cells by heterologous expression and subsequent immunoprecipitation (Figure 4C). These results demonstrate that CFAP77 is important for the stable integration of MIPs, particularly TEKTL1, into mature spermatozoa.

### 3.5 CFAP77‐Interacting Protein TEKTL1 Affects Sperm Velocity Independent of CFAP77

We next questioned if and how TEKTL1 contributes to the male fertilization process. We have previously generated *Tektl1* KO males and reported that they had normal fertility with normal spermatogenesis [38]. However, although the male fertility is normal, sperm motility could be compromised. To explore this possibility, we measured the sperm motility of *Tektl1* KO males. We found that the absence of TEKTL1 did not affect the percentages of motile or progressively motile spermatozoa (Figure 4D). In contrast, VCL decreased in *Tektl1* KO males in 10 min and 120 min of incubation, and VSL decreased in 120 min of incubation (Figure 4E and Movie S1). VAP of *Tektl1* KO spermatozoa also showed a decreasing trend, but it was not significant. By performing immunohistochemistry on the testicular section of *Tektl1* KO mice, we determined that CFAP77 is present in the flagella of *Tektl1* KO testes (Figure 4F). These results suggest that TEKTL1 is involved in sperm motility independent of CFAP77. As the motility reduction of *Tektl1* KO spermatozoa is not as severe as that of *Cfap77* KO spermatozoa, the reduced sperm motility of *Cfap77* KO mice cannot be explained only by the loss of TEKTL1.

## 4 Discussion

As more MIPs were identified by cryo‐EM, their functions in mammalian male reproduction are expected to be explored. In the current study, we confirmed that CFAP77 is present in mature spermatozoa of mice and humans (Figure 1B–D), and it is likely serving as a MIP in mice, as it was found in the axoneme‐associated protein fraction (Figure 1C). Mouse *Cfap77* is predominantly expressed in the testis with weaker expression in the lung (Figure S1C), which is likely an indication of CFAP77's presence in the ciliary axoneme. In the testis, *Cfap77* exhibits high mRNA levels in round spermatids, where the sperm axoneme proceeds to be assembled [39]. To examine the role of CFAP77, we generated *Cfap77* KO mice, which showed significantly declined male fertility relative to their WT counterpart (Figure 2D). While no overt abnormalities were found in male reproductive organs or spermatozoa (Figure 3A), sperm motility was severely impaired in *Cfap77* KO males (Figure 3B,C).

Our interaction study revealed that CFAP77 interacts with 6 proteins in the testis (Log_2_FC > 10, *p* value < 0.05), which are TEKTL1, SPMIP8, CENPJ, EPN1, LGALS8, and CSNK1G3 (Figure 4A). Among these proteins, TEKTL1 is the only protein that significantly decreased in *Cfap77* KO cauda epididymal spermatozoa (Figure 4B). SPMIP8 is an MIP resident at A09–A12 protofilaments along the sperm axoneme at a periodicity of 48 nm [13]. Because the previous cryo‐EM study did not show the direct interaction between CFAP77 and SPMIP8 [13], further analyses are required to understand their relationship. CENPJ is localized in both mother and daughter centrioles in ciliary cells [40]. While gene ablation of *CENPJ* causes the shortening of primary cilia in neuronal cells [41, 42], conditional KO of *Cenpj* in mice results in abnormally prolonged ciliary length in ependymal multiciliated cells [43]. It is possible that CFAP77 contacts CENPJ in centrioles during flagellar formation. Overall, while CFAP77 is associated with TEKTL1, CFAP77 interacts with other proteins, and their functional roles require further investigation.

To understand whether the decreased motility of *Cfap77* KO spermatozoa could be explained by the loss of TEKTL1, we analyzed sperm motility and CFAP77's localization in *Tektl1* KO mice. We found that the ablation of TEKTL1 resulted in a partial reduction of sperm velocity parameters without loss of CFAP77 (Figure 4E,F), suggesting that TEKTL1 regulates sperm motility independently of CFAP77. Given its localization within the microtubules, TEKTL1 may regulate sperm motility by contributing to the maintenance of microtubule stiffness. Given that TEKTL1 is lost in *Cfap77* KO spermatozoa, it is possible that CFAP77‐independent motility control by TEKTL1 partially contributes to the reduced motility of *Cfap77* KO spermatozoa.

In *Trichomonas vaginalis* and *Tetrahymena thermophila*, CFAP77 was identified as an outer junction protein that maintains axonemal stability [19, 44]. In *Tetrahymena thermophila*, KO of CFAP77A/B (two orthologs of CFAP77 in *Tetrahymena thermophila*) leads to the formation of unstable B‐tubule, subsequently causing decreased ciliary length and impaired ciliary motility. Consistently, damages in the outer junctions may contribute to the reduced sperm motility in *Cfap77* KO mice. In WT and *Tektl1* KO mice, the percentages of progressive spermatozoa out of motile spermatozoa did not change during incubation in a capacitation medium (Figure S4A). In contrast, this ratio significantly dropped for *Cfap77* KO spermatozoa subsequent to incubation (Figure S4B). Therefore, it is possible that sperm movement exerts a force on microtubule doublets, which may damage the integrity of the outer junction and may cause a large decline in progressive motility during incubation.

Upon preparing this manuscript, we found that another group independently concluded that CFAP77 is important for sperm motility by maintaining the B‐tubule arrangement at the outer junction [45]. By employing transmission electron microscopy, their study revealed that more open B‐tubules were observed in the axoneme of cilia and spermatozoa of *Cfap77* KO mice, complementing the structural data that were lacking in our research. Moreover, they described that *Cfap77* KO results in complete infertility, but not subfertility, as we found. This could be caused by the difference in the genetic background, as experiments were performed with the B6D2 hybrid background in this study, while C57BL/6 inbred mice were used in another group. Generally, mice with the B6D2 hybrid background exhibit higher litter size compared with their counterparts from the C57BL/6 background, and this higher litter size is attributable to both female and male factors [46, 47]. B6D2 hybrid females ovulate more oocytes from superovulation [48, 49], and those oocytes have better tolerance to sperm injection and high salt environment [50], indicating B6D2 oocytes have higher oocyte quality as opposed to C57BL/6 oocytes. On the male side, B6D2 hybrid mice have higher sperm motility than C57BL/6 mice [27, 51, 52]. The discrepancy in oocyte quality and sperm motility of mice from the two genetic backgrounds could explain the less severe phenotypes observed in this study.

In summary, our study found the important role of CFAP77 in assuring sperm motility and male fertility in mice. As CFAP77 is evolutionarily conserved in multiple species, it is possible that its functions in humans are similar to those described in this study. However, as the roles of *CFAP77* variations in human male infertility remain unclear, further investigation is warranted. Further studies on MIPs may boost the comprehension of MIP‐dependent motility control mechanisms in spermatozoa.

## Author Contributions

**Haoting Wang**: conceptualization, investigation, formal analysis, manuscript preparation. **Anh Hoang Pham**: investigation, formal analysis, manuscript preparation. **Mengjiao Luo**: investigation, manuscript preparation. **Masahito Ikawa**: conceptualization, supervision, funding acquisition, manuscript preparation. **Haruhiko Miyata**: conceptualization, investigation, supervision, funding acquisition, manuscript preparation.

## Funding

This research was supported by the Ministry of Education, Culture, Sports, Science and Technology/Japan Society for the Promotion of Science KAKENHI grants (JP21H05033 and JP23K20043 to M.I., and JP23K18328 and JP25K02773 to H.M.); Japan Science and Technology Agency (JST) grants (JPMJCR21N1 to M.I. and JPMJFR211F to H.M.); Japan Agency for Medical Research and Development (AMED) grant, JP23jf0126001 to M.I.; Eunice Kennedy Shriver National Institute of Child Health and Human Development (R01HD088412 to M.I.).

## Conflicts of Interest

The authors declare no conflicts of interest.

## Supporting information




**Figure S1**: Conservation, comparison, and expression profiles of *Cfap77*.

**Table S1**: The sequences of gRNAs and primers used in this study

**Table S2**: Antibodies used in this study

**Table S3**: Immunoprecipitation‐mass spectrometry analysis of CFAP77

**Table S4**: Proteomic analysis on *Cfap77* KO spermatozoa

**Movie S1**: Motility of WT, *Cfap77* KO, and *Tektl1* KO spermatozoa
