# Supplementary material for: Microtubule Inner Protein CFAP77 Contributes to Sperm Motility and Male Fertility in Mice
Source: Andrology. 2025 Nov 29;14(5):1240–8. doi: 10.1111/andr.70152 (PMC13002332; doi:10.1111/andr.70152)
Supplement: Supplementary file 1 — Figure S1: Conservation, comparison, and expression profiles of Cfap77. [file ANDR-14-1240-s001.pdf]

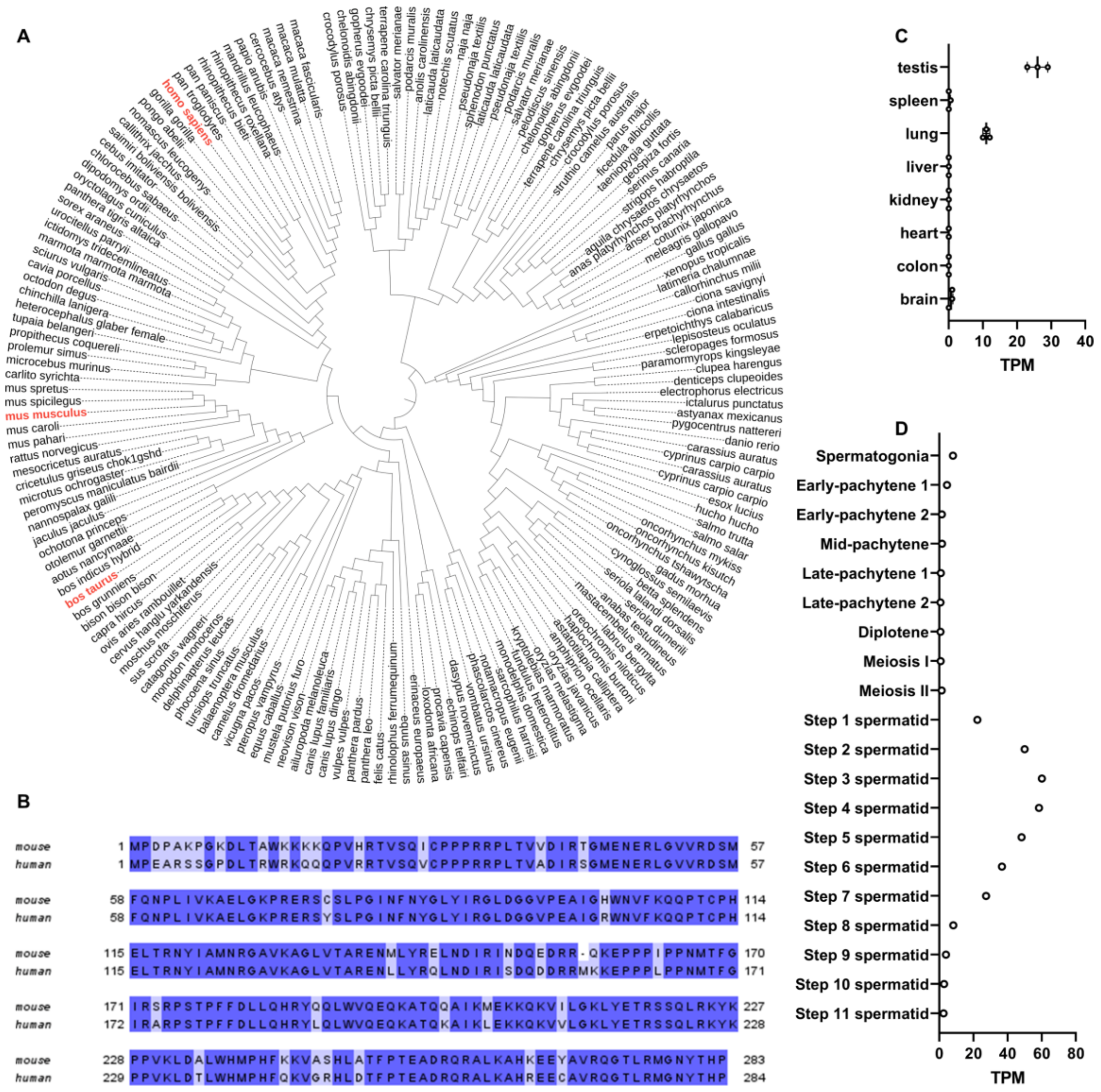

Figure S1. Conservation, comparison, and expression profiles of *Cfap77*

(A) Gene conservation of *Cfap77* in multiple species. (B) Comparison of amino acid sequences of CFAP77 in mice and humans. (C) Expression of *Cfap77* in multiple organs of mice. TPM stands for transcripts per million. (D) Expression profile of *Cfap77* in mouse testicular germ cells.

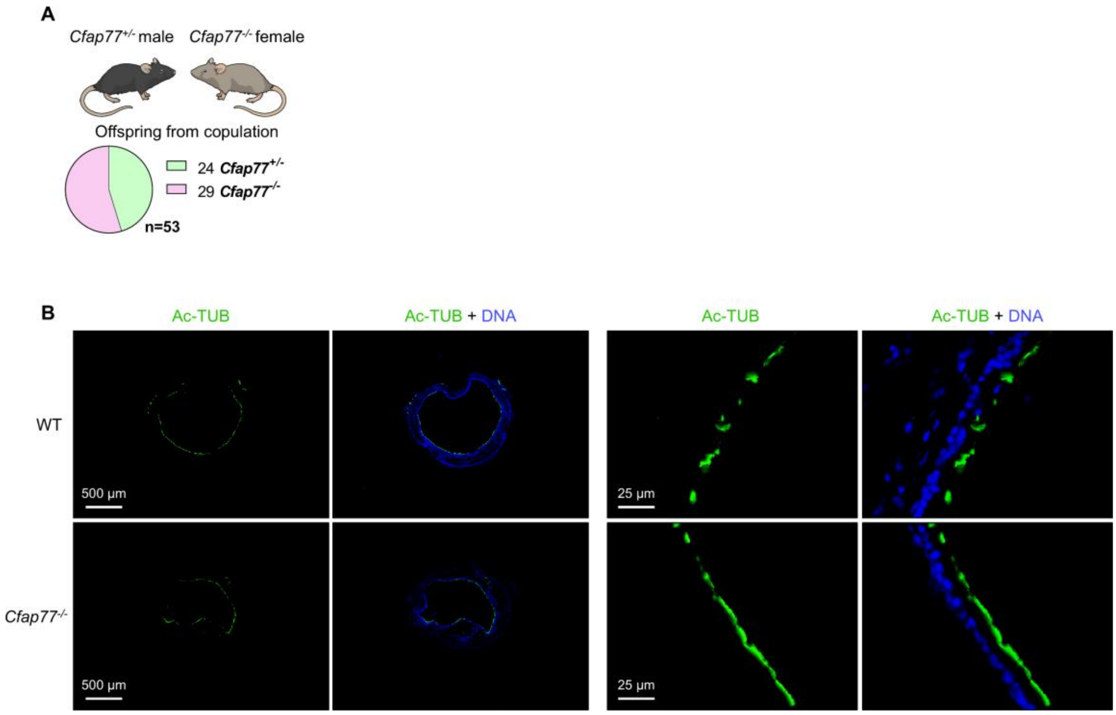

Figure S2. No overt abnormalities were found in the ciliogenesis of *Cfap77* KO trachea

(A) Offspring derived from one *Cfap77*$^{+/-}$ male mated with two *Cfap77*$^{-/-}$ females. The average litter size of two *Cfap77*$^{-/-}$ females was 6.6±1.8 (the number of liters = 8). (B) Immunohistochemistry using an anti-acetylated-tubulin (Ac-TUB) antibody to label tracheal cilia.

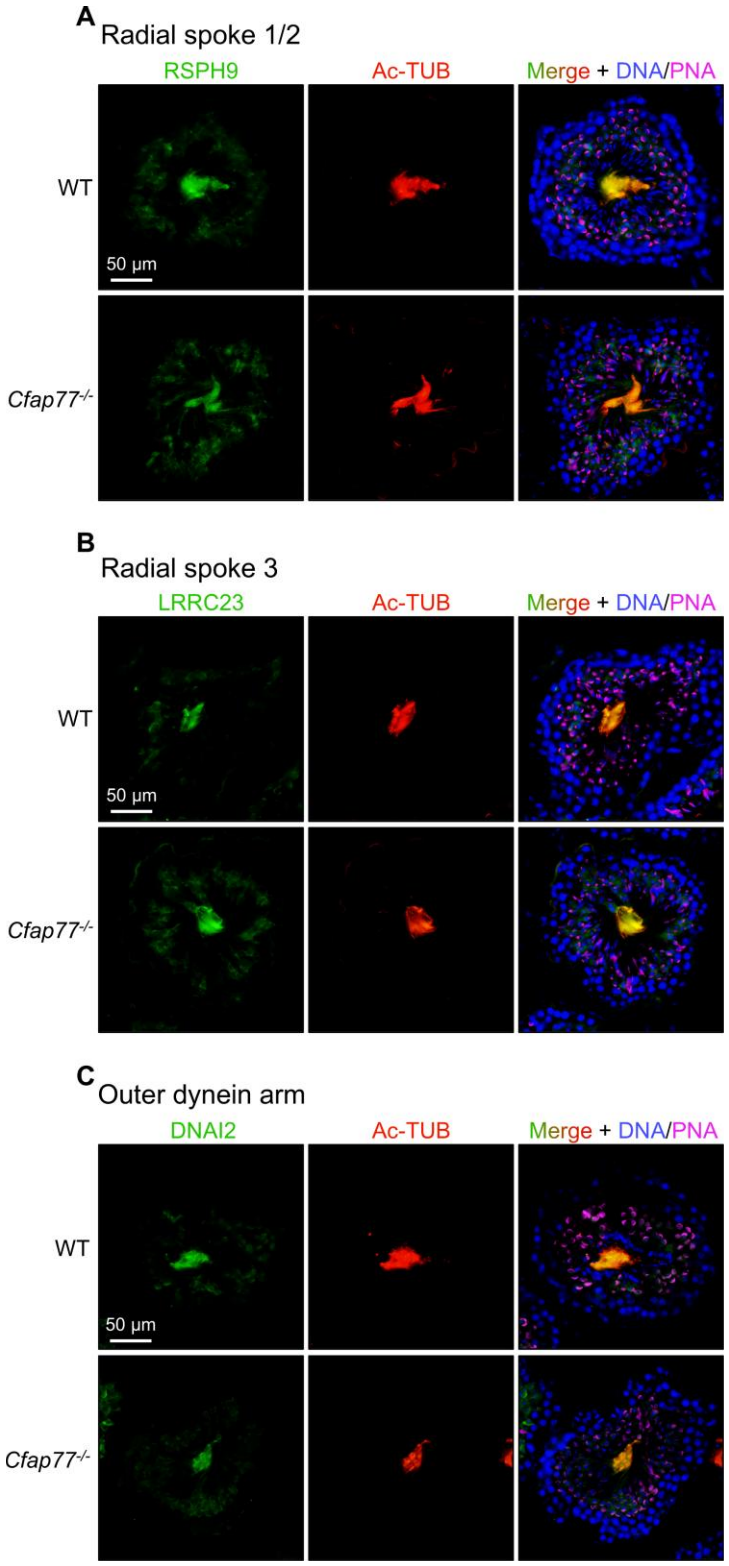

Figure S3. Immunohistochemistry of axonemal proteins in *Cfap77* KO testes

(A - C) Immunohistochemistry that labeled RSPH9 (A), LRRC23 (B) and DNAHI2 (C) in WT and *Cfap77* KO testicular sections. An anti-acetylated tubulin (Ac-TUB) labels sperm flagella while PNA labels the acrosomes.

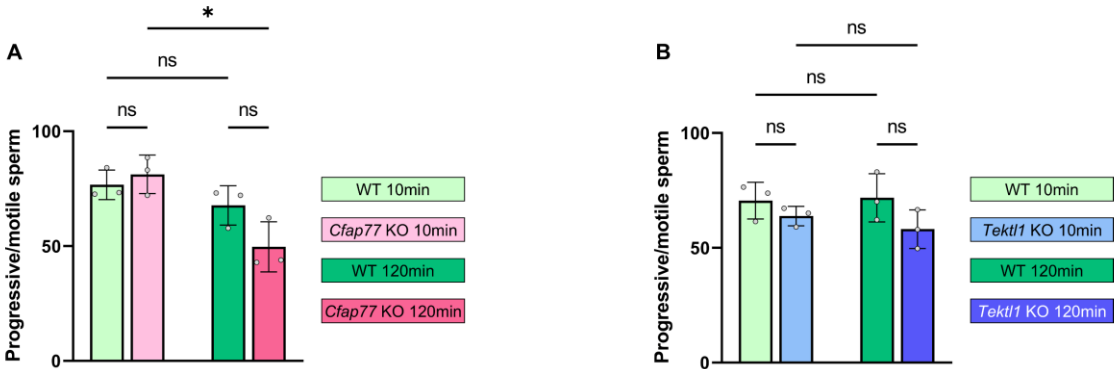

Figure S4. The percentages of progressive spermatozoa out of motile spermatozoa

Progressive spermatozoa/motile spermatozoa ratios from Figure 3B and 4D for each individual mouse were calculated.

Table S1. The sequences of gRNAs and primers used in this study

Table S2. Antibodies used in this study

Table S3. Immunoprecipitation-mass spectrometry analysis of CFAP77

Table S4. Proteomic analysis on *Cfap77* KO spermatozoa

Movie S1. Motility of WT, *Cfap77* KO, and *Tektl1* KO spermatozoa
